# Sleep characteristics in patients with substance use disorder after detoxification treatment: self-report and actigraphy data

**DOI:** 10.1192/j.eurpsy.2022.2093

**Published:** 2022-09-01

**Authors:** M. Vetrova, K. Rybakova, O. Goncharov, E. Krupitsky

**Affiliations:** 1 Pavlov University, Institute Of Pharmacology, Saint Petersburg, Russian Federation; 2 Bekhterev National Medical Research Center for Psychiatry and Neurology, Department Of Addictology, Saint Petersburg, Russian Federation

**Keywords:** substance use disorder, Russia, sleep, actigraphy

## Abstract

**Introduction:**

Sleep problems are common in patients with substance use disorders (SUD) and have been related to poor treatment outcomes. Little is known about the sleep characteristics in patients with opioid and alcohol use disorders after detoxification program.

**Objectives:**

To compare sleep quantitative and qualitative characteristics between patients with opioid and alcohol use disorders.

**Methods:**

This is a secondary data analysis of the longitudinal data from the observational study in St. Petersburg, Russia. The sample included 75 patients (22.7% female) who received detoxification treatment for alcohol (n=49) or opioid (n=26) withdrawal. Participants completed the Pittsburgh Sleep Quality Index (PSQI) and underwent daily wrist actigrahy.

**Results:**

Good internal consistency was demonstrated for self-report and actigraphy data (*r* =-0,405, p<0,01). Sleep duration and sleep onset latency were not different between alcohol and opioid groups (5.7 vs. 6.1 hours; 74 vs. 65 minutes, respectively) based on self-report data. The majority of the patients (57-100%) had sleep complaints and low quality of sleep after detoxification completion (at baseline). In both groups, the mean PSQI score had a tendency to decrease, representing better sleep quality, over the 1-week following detoxification program completion (from 12 at baseline to 10 at 1-week in alcohol group; from 13 to 12 in opioid group, *p*<0,001).

**Conclusions:**

The findings show that sleep characteristics are similar in patients with different SUD and insomnia symptoms are prevalent after detoxification, suggesting the rationale for sleep assessment before hospital discharge. Despite the positive changes in sleep quality over 1-week abstinence, patients might benefit from the therapeutic sleep interventions.

**Disclosure:**

This work was financially supported by a research grant from Russian Foundation for Basic Research, 18-013-00481.

